# Goji Berries Supplementation in the Diet of Rabbits and Other Livestock Animals: A Mini-Review of the Current Knowledge

**DOI:** 10.3389/fvets.2021.823589

**Published:** 2022-01-31

**Authors:** Stella Agradi, Susanna Draghi, Elisa Cotozzolo, Olimpia Barbato, Marta Castrica, Alda Quattrone, Majlind Sulce, Daniele Vigo, Laura Menchetti, Maria Rachele Ceccarini, Egon Andoni, Federica Riva, Maria Laura Marongiu, Giulio Curone, Gabriele Brecchia

**Affiliations:** ^1^Department of Veterinary Medicine, University of Milan, Lodi, Italy; ^2^Department of Agricultural, Food and Environmental Sciences, University of Perugia, Perugia, Italy; ^3^Department of Veterinary Medicine, University of Perugia, Perugia, Italy; ^4^Department of Health, Animal Science and Food Safety “Carlo Cantoni”, University of Milan, Milan, Italy; ^5^Faculty of Veterinary Medicine, Agricultural University of Tirana, Tirana, Albania; ^6^Department of Agricultural and Food Sciences, University of Bologna, Bologna, Italy; ^7^Department of Pharmaceutical Sciences, University of Perugia, Perugia, Italy; ^8^Department of Veterinary Medicine, University of Sassari, Sassari, Italy

**Keywords:** *Lycium barbarum*, nutraceutical, reproductive and productive performance, meat quality, immune system, metabolism, Chinese traditional medicine, polysaccharides

## Abstract

In the last decades, several nutraceutical substances have received great attention for their potential role in the prevention and treatment of different diseases as well as for their beneficial effects in promoting the health of humans and animals. Goji berries (GBs) are the fruit of *Lycium barbarum* and other species of *Lycium*, used in traditional Chinese medicine, and they have recently become very popular in the Occidental world because of their properties, such as anti-aging, antioxidant, anticancer, neuroprotective, cytoprotective, antidiabetic, and anti-inflammatory activities. These effects are essentially evaluated in clinical trials in humans; in experimental animal models, such as mice and rats; and in cell lines in *in vitro* studies. Only recently has scientific research evaluated the effects of GBs diet supplementation in livestock animals, including rabbits. Although studies in the zootechnical field are still limited and the investigation of the GB mechanisms of action is in an early stage, the results are encouraging. This review includes a survey of the experimental trials that evaluated the effects of the GBs supplementation on reproductive and productive performances, immune system, metabolic homeostasis, and meat quality principally in the rabbit with also some references to other livestock animal species. Evidence supports the idea that GB supplementation could be used in rabbit breeding, although future studies should be conducted to establish the optimal dose to be administered and to assess the sustainability of the use of GBs in the diet of the rabbit.

## Introduction

There is a growing interest worldwide in the development of nutraceutical products that could ensure potential health benefits and greater life quality (1). The biologically active compounds included in the nutraceuticals may have a role in the prevention and treatment of several diseases not only in humans but also in animals (2–5).

**Figure 1:**
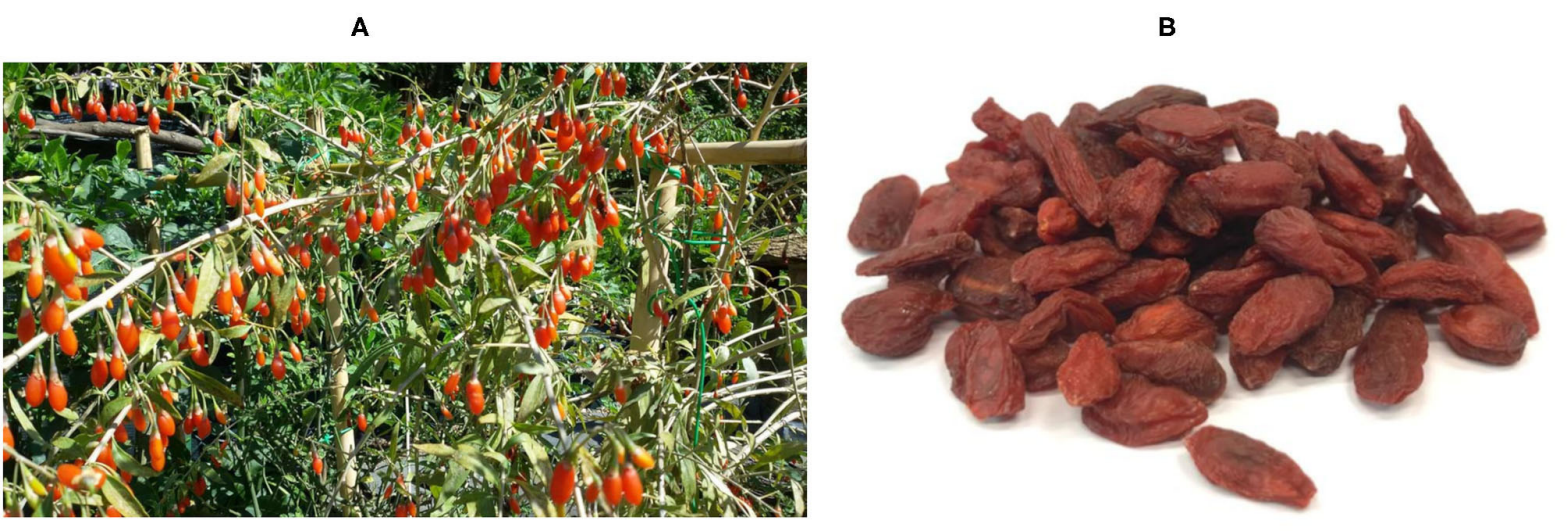
*Lycium barbarum* plant **(A)** is a deciduous shrub 1–3 m high with lanceolate leaves. The berries are orange to dark red, oblong, measure up to 2 cm and have a bitter-sweet taste (by courtesy of Mrs. Gilberta Dal Porto). The fruits of Goji plant are usually commercialized as dried berries (9) **(B)**.

Recently, the interest in medicinal herbs and plant extracts/metabolites has increased, both among the general public and researchers worldwide (6–8). Goji berries (GBs) are the fruit of *Lycium barbarum* (Figure 1A) and other species of *Lycium* which are becoming more and more famous in the Western countries because of their properties, in particular the anti-aging ones (9). This interest also comes from the absence or the negligible presence of side effects in comparison with traditional pharmacological therapies (10). Goji berries are a nutraceutical product because of their benefits for human health, such as immunomodulatory (11), anticancer (12), anti-aging (13), neuroprotective (14), gastrointestinal protective (15), cytoprotective (16), antioxidant (17), antidiabetic (18), anti-inflammatory (19), visual protective (20), and radiation protective effects (21). Most of the evidence on the beneficial effects of GB consumption derives from clinical trials in humans, experimental studies in laboratory animals, and *in vitro* trials (22, 23). On the contrary, data on GBs' effects on livestock animals, including rabbits, are not very numerous (24, 25).

The rabbit represents not only a livestock animal and a pet, but also an experimental animal model that is currently used to study a wide range of physiological processes related to reproduction (26–29), digestion (30–32), metabolic homeostasis (33–35), and immunology (36). The supplementation of the rabbit's diet with GBs may have different beneficial effects on rabbit breeding, such as: (i) increase of the reproductive and productive performances; (ii) reduction of the use of antibiotics and mortality as well as infectious diseases due to the growth of a beneficial intestinal microbiota, which in turn stimulates the immune system; (iii) improvement of the health status and welfare; (iv) improvement of the meat quality which can be used as a functional food for humans (37, 38). These effects could also be obtained in other livestock animal species such as swine (24, 39), poultry (25, 40), and fish (41, 42).

The present review summarizes the most relevant literature on the effects and mechanism of action of GB supplementation in the diet of livestock animals, especially in rabbits.

## Literature Research

The literature search was performed using the following databases: PubMed, Web of Science, CAB Abstracts Archive, and Google Scholar (consulted till September 2021). For the search, the keyword terms used included: rabbit, *Oryctolagus cuniculus*, livestock, chicken, broiler, swine, sheep, goat, cattle, cow, beef, horse, fish, *L. barbarum, Lycium*, wolfberry, Goji, Goji berry, reproduction, reproductive performance, production, productive performance, immune system, immunomodulation, metabolism, metabolic homeostasis, and meat quality, in different combinations. Only English language papers were considered. Over 100 papers had been analyzed but 27 were selected as fully satisfying the purpose of this review. These papers were stratified based on the main activity investigated, the species, and the experimental model (Table 1).

**Table 1:** Summary of the main experimental studies investigating *Lycium* plant pro-activities on livestock animals.

**Pro-activity on**	**Species**	**Method**	**References**
Reproductive performance	Rabbit	*In vivo*	(43–45)
	Swine	*In vitro*	(46)
		*In vivo*	(47)
	Goat	*In vitro*	(48)
Productive performance	Rabbit	*In vivo*	(45, 49, 50)
	Chicken	*In vivo*	(25, 40, 51)
	Swine	*In vivo*	(24)
	Sheep	*In vivo*	(52)
	Fish	*In vivo*	(42, 53)
Immune system	Rabbit	*In vivo*	(50, 54)
	Chicken	*In vivo*	(42, 53)
	Swine	*In vivo*	(24)
	Sheep	*In vivo*	(52)
	Fish	*In vivo*	(42, 53)
Metabolic homeostasis	Rabbit	*In vivo*	(44, 55)
	Swine	*In vivo*	(39)
Meat quality	Rabbit	*In vivo*	(50, 56, 57)
	Swine	*In vivo*	(39)
	Sheep	–^a^	(58, 59)
	Cow	–^a^	(60)
	Camel	–^a^	(61)
	Horse	–^a^	(62)
	Fish	–^a^	(63, 64)

*Many studies used Goji berry or its derivative products, while just a few involved Goji leaves or bark root*.

a *These studies performed microbiological and/or quality meat assays*.

## General Information on Composition, Pharmacology, and Safety of Goji

Goji comes from the Chinese “gouqi” (9). Its first mention in Chinese lore is dated 2800 B.C., associated with a mythological Chinese sovereign to whom the book “The Divine Farmer's Herb-Root Classic” is attributed (65). Its use in traditional Chinese medicine is testified by different records (66). Moreover, root bark and leaves of *L. barbarum* have been known for centuries in Eastern countries for their properties (67). Several species and varieties of *Lycium* are cultivated for berry production, like *L. barbarum aurantiocarpum* and *Lycium chinense pataninii*.

Goji berries are the most used part of the *Lycium* plant (Figure 1B), and their main activities, according to traditional Chinese medicine, are on the liver and kidneys (65). Goji berries contain abundant bioactive molecules, with more than 200 different identified components (68, 69). *Lycium barbarum* polysaccharides (LBPs) are the most investigated components. They include a wide group of water-soluble molecules, constituting 5–8% of the dried berry (70). *Lycium barbarum* polysaccharides contain six different monosaccharides (mainly xylose and glucose), galacturonic acid, and 18 different amino acids (71). Moreover, GBs are also considered a good source of both carotenoids, with zeaxanthin as the main fraction, and polyphenols, mainly quercetin and kaempferol (72). Different studies have demonstrated that the health benefits deriving from the consumption of GBs are due to a structurally varied range of molecules, which includes all the abovementioned (55, 73, 74).

The 2010 Chinese Pharmacopeia reported the root of *L. barbarum* or *chinense* (Cortex Lycii) to be useful in the treatment of diabetes mellitus, coughs, hematemesis, hypertension, night sweats, and ulcers (75). More than 50 different phenolic amides (76) and two new molecules with interesting anti-inflammatory effects (77) have been recently identified in *L. chinense* root bark. Compared to the fruit and leaves, the root is characterized to have the lowest amount of compounds identifiable, although its extract has stronger antioxidant activity than berries and leaves (67).

Leaves of *L. barbarum* have been used in Chinese traditional medicine for the treatment of liver diseases and the improvement of eyesight (78). Their main constituents are polysaccharides, phenolic acids, flavonoids, coumarins, carotenoids, and alkaloids (79). The antioxidant activity of leaves has been demonstrated to be higher compared to berries but lower than the bark root of *L. barbarum* (67).

To date, there is no evidence of the toxicity of *L. barbarum* in the scientific literature, and the Asian traditional culture considers it and its derivatives to be highly safe at different dosages (71), whereas only a few studies reported minor adverse effects and mild toxicity (80, 81).

## Goji Effects on Reproductive Traits

The rabbit is a livestock animal in which productive efficiency is strongly correlated with reproductive performance (34). Furthermore, the rabbit is also a useful animal model to study the physiological processes linked to the reproduction of both males (82–84) and females (85–87). In rabbit breeding, one of the most critical points is the maintenance of the energy balance during the reproductive cycle; the unbalance has consequences on both profitability of the farm and animal welfare (9, 88, 89). The adoption of new feeding strategies with the use of nutraceutical substances could be a good way to improve these aspects. The possible use of GB to improve reproductive parameters has been investigated in a few studies on rabbit does, while currently there is no published data about its effect on male rabbits' reproductive performance.

A first study, during the 80s, evaluated the effect of *L. chinense* on female rabbits (43). The crude extract of leaves induced the ovulation in the adult does by intravenous administration; however, the crude extracts of root bark and berries were not able to produce the same effect (43). More recently, New Zealand White nulliparous rabbit does were fed with diets supplemented with 1 and 3% of GBs during pregnancy and lactation (44). According to the dose, the supplementation seemed to modulate the energy homeostasis during the reproductive cycle; 1% inclusion of GBs improved insulin resistance, while 3% caused excessive fattening and reduced insulin sensitivity (44). Different dose-related effects have been shown in a third study that had a similar protocol and the same category of animals (45). In this investigation, GBs at 1% enhanced the receptivity (measured by the vulva color evaluation) and induced a higher peak of 17β-oestradiol. The group fed with 3% GB-supplemented diet, instead, showed no changes in receptivity but a delay of the 17β-oestradiol peak compared to the group fed with the standard diet. Thus, GBs could play a role in rabbit reproduction improving energy balance and interfering with the hormonal picture. The correct formulation, however, has to be modulated and further investigated with a larger sample size.

In other species, LPBs' protective effect against damage by substances and pathologies on the male reproductive system has been evaluated. This effect has been correlated to their antioxidant activity due to the increase of the antioxidant enzyme potential and the reduction of reactive oxygen species production. These studies have been conducted mainly on laboratory animals (90–92). Positive effects have also been observed on the *in vitro* centrifugation process in human asthenospermia sperm (93). The pro-sexual activity of LPBs was demonstrated on normal and sexually inhibited rats, probability through its effects on neurogenesis (94). In boars (47) and Cashmere goats (48), the semen quality was improved when LBPs were administered alone in the diet or in the base extender of semen samples in association with *Laminaria japonica* polysaccharide, respectively. The positive effect of LPBs on ovarian injuries induced by repeated superovulation has been investigated in mice (95). Finally, research conducted on porcine oocytes and two-cell murine embryos has demonstrated the aptitude of LPBs to act as a cryoprotectant thanks to its antioxidant activity (46, 96).

The effects of GB on the reproductive system are still very limited in the livestock species, although the first results in rabbits, laboratory animals, and some *in vitro* trials are really encouraging.

## Goji Effects on Productive Performances

Goji berry could have a significant effect on the productive performance of livestock animals by both improving growth parameters and reducing economic losses (53).

In cuniculture, a first investigation involved New Zealand White nulliparous does fed with 0, 1, or 3% dried GBs supplemented diets from 30 days before artificial insemination until weaning (49). Afterwards, their litters were fed with the corresponding mothers' diet until slaughtering. Compared to the control group, the does fed with GBs showed a lower feed intake during non-pregnant and lactating periods, while the 3% GB group consumed the highest quantity of fed during pregnancy. A dose-dependent effect was also found for milk production: 1% GB group showed the highest milk yield, while the 3% GB group the lowest. The authors hypothesized that in the 1% GB group the extra energy supplied by the berries was successfully addressed to milk production, while in the 3% GB group the berries' anti-nutritional activity supplemented at high doses resulted in a negative effect. This finding could also be explained by a reduction in the insulin sensitivity in lactating rabbits of the 3% GB group (44). Moreover, the 1% GB group had lower pre-weaning mortality, higher litter size at 18 days and at weaning, and higher litter weight at day 18; conversely, the 3% GB group had no significant differences compared to the control group. These results could be ascribed to the higher milk yield of the 1% GB does and to hypothetical changes in immunomodulatory properties of milk. However, all rabbits receiving GB in the fattening period exhibited higher body weight (BW) at slaughter and lower feed conversion rate (49). This result was not in agreement with the study of Liu et al. (50). These authors evaluated the effects of supplementations with 0.1, 0.2, and 0.3% of LBPs for 30 days on Rex rabbits of both sexes but did not find differences on their slaughter performance (50). The different protocol and diet composition could explain the inconsistency between the results. Finally, another study has recently evaluated the productive performance of nulliparous New Zealand White rabbit does (and their litters) fed with a standard and 1 or 3% GBs supplemented diet (45). It confirms the positive effect of GB supplementation on weight and litter size at weaning but the BW at slaughter was not investigated (49). Specific feed formulations could be developed and validated including different GB doses according to the growing phase.

Regarding other zootechnical species, two experiments showed a positive effect of LBPs on the growth performance of broiler chickens. In particular, the diet supplemented with 0.2% LBP improved BW, average daily gain (ADG), and average daily feed intake (ADFI), while 0.4% supplemented diet improved ADFI and feed conversion rate (25, 40). In laying hens, a reduction of yolk cholesterol level has been obtained using a supplementation of 2% *L. barbarum* leaves for 8 weeks, but no other differences were found in the productive parameters (51). The effect of diets supplemented with different levels of LPBs (0, 0.1, 0.2, 0.4, and 0.6%) administered to crossed weaned piglets have also been investigated (24). Due to an increase in palatability and digestibility, 0.4% LBPs diet improved ADG and ADFI and decreased diarrheal incidence during weaning. Similar results were also obtained in a study on lambs fed for 60 days with a standard diet or supplemented with 0.1% of *L. barbarum* and *Astragalus membranaceus* 2:1 (52). Significant improvement in ADG and feed to gain ratio were shown, demonstrating LBPs' ability to enhance growth performance also in neonatal polygastrics. In pisciculture, two studies used *L. barbarum* extract (42, 53). In a first study, farmed fish fed a 0.2% supplemented diet showed a positive effect on growth performance that could be ascribed to its stimulating activity on the digestive enzymes and/or positive changes in the gut microbiota (53). Conversely, a supplementation with 2% GBs had null or negative results, probably due to the inhibition of specific gut microbial communities (53). A second investigation showed a significant positive effect on growth parameters of hybrid grouper fed high lipid diets with *L. barbarum* extract supplementation (0.2 and 1%) (42).

Studies regarding the effect of Goji-supplemented diets on productive performance are transversally encouraging in different livestock species. The correlation with microbiota modifications should be further investigated as induced changes in the gastrointestinal microbiota and digestive enzymes activities have been shown in different studies conducted on zootechnical species (24, 25, 52).

## Goji Effects on the Immune System

Goji berries' immunomodulatory activity has been greatly investigated during the last two decades, especially correlated to LPBs. Clinical studies have been conducted mainly on humans and laboratory animals or *in vitro*, as reviewed by Xiao et al. (97) and Kulczyński et al. (73). Many studies investigated the mechanism of action of LPBs, aiming to implement GB use in human medicine. Thus, GB supplementation could also have a positive effect on the immune system of livestock animals and improve not only productive performances but also their welfare state. Moreover, antimicrobial resistance is becoming a worldwide recognized threatening phenomenon (98). One of the main suggested measures incentivized by the European Union (99, 100) to contrast antimicrobial resistance consists of replacing antimicrobials with alternative treatments to improve animal health and reduce antibiotic usage. Nevertheless, the studies conducted so far on GB use in zootechnical species with the investigation of immunomodulating effect are still limited.

Liu et al. (50) found a trend for a dose-dependent positive effect of LBP supplementation on the immune organ development (thymus and spleen) of 40-day-old Rex rabbits; however, the differences were not statistically significant (50). Another study in rabbits investigated LBPs' effect on renal function and inflammatory reaction caused by diabetes mellitus-induced nephropathy (54), showing positive preventive and treatment effects on both renal function failure and renal cortex inflammatory reaction (54).

In broiler chickens, the supplementation of standard diet with 0, 0.1, 0.2, and 0.4% of LBPs could improve specific immune response toward infections and enhance antibody production in a dose-dependent way (25). Another study on broilers showed that LBPs could regulate lymphocyte proliferation and inflammatory cytokine expression both *in vivo* and *in vitro* (40). Immunoglobulin increase was also found in Chuanzhong black lambs fed 60 days with a combination of *L. barbarum* and *A. membranaceus* (52). In piglets, 0.4% LBP-supplemented diet could significantly decrease the diarrheal incidence and increase serum IgG and IgM concentrations (24). *Lycium barbarum* extract was supplemented for 8 weeks at different concentrations (from 0.05 to 2%) in two studies involving different species of Chinese farmed fish (42, 53). A liver protective effect of *L. barbarum* extract against high-lipid diets has been demonstrated, probably due to its ability not only to increase hepatic antioxidant enzyme activity and their genes' expression but also to modulate the immune system by lowering the hepatic inflammatory response and apoptosis (42). Although the second study obtained less significant results, it suggested that *L. barbarum* extract could improve several non-specific immune parameters and increase the fish's immune system efficiency (53).

The studies conducted so far seem to justify the importance that Chinese traditional medicine attributed to Goji. However, further investigations, both *in vivo* and *in vitro*, will be needed because GB could be useful in reducing the use of antibiotics.

## Goji Effects on Metabolic Homeostasis

Rabbits are a good animal model to study the effects of different nutraceutical substances on metabolism (89, 101–103). Goji berry has the ability to modulate the metabolism of different substances in the organism, and considering its known lack of toxicity it could be employed in the prevention and/or treatment of various pathologies (71). Its hypoglycemic effect has received particular attention because of its possible use in the treatment of diabetes mellitus in humans (70). Both experimental studies on laboratory animals and clinical studies on diabetic patients have been conducted as reviewed by Amagase et al. (71).

There are a few studies regarding the effects of GB on the metabolic homeostasis in rabbits. In a first study, 35 adult rabbits with alloxan-induced hyperglycemia were fed three different GB preparations (fruit water decoction, crude LBPs, and purified LBPs) for 10 days (55). In the GB-treated groups, a significant reduction in glycemia, decrease in lipemia, and increase in high-density lipoprotein cholesterol were found. Thus, it was demonstrated that all treatments could produce significant hypoglycemic and hypolipidemic effects in rabbits. On the other hand, a study by Menchetti et al. used 75 New Zealand White nulliparous does to investigate the effects of GB, supplemented at 1 and 3%, on the energy homeostasis during the reproductive cycle (44). As mentioned above, the maintenance of the positive energy balance during pregnancy is the key for both profitability and animal welfare in the rabbitries (9, 88, 89). The main reason for the negative energy balance in rabbit does is the overlapping of pregnancy and lactation, causative of a fertility reduction (33). In the early lactation, as confirmed by the control group of this experiment, there is usually both a condition of insulin resistance and an increase of non-esterified fatty acids (44). The 3% GB diet exacerbated the condition of insulin resistance and significantly increased BW and Body Condition Score (BCS). Conversely, the 1% GB diet improved insulin sensitivity. Through a multivariate approach, the authors also analyzed the interactions between metabolic hormones and body conditions demonstrating that as the percentage of GBs supplementation increases, leptin, BW, and BCS of rabbits tend to increase. These findings suggest that high doses of berries could cause excessive fattening and therefore negatively affect performance, as already demonstrated by Andoni et al. (45). The effects on metabolism can have repercussions not only on productive and reproductive performances but also on the welfare of rabbits.

Regarding other zootechnical species, a study conducted on Pietrain pigs evaluated the ability of *L. barbarum* to reduce the metabolic negative effects induced by pre-slaughter stress caused by the use of an electric prod (39); 1% dried GBs supplemented diet fed for 7 days did not affect serum lactic acid concentrations. However, glycemia had a significant decrease compared to the control group, while glycogen concentration in the liver showed a significant increase. Thus, *L. barbarum* was demonstrated to possess good restoring antioxidant ability in stressed pigs and may be used in the prevention of pale, soft, and exudative meat in the pork industry.

Information about modifications of the metabolism induced by GB in livestock animals is still very limited. However, first results suggest that its use during critical phases, such as gestation or pre-slaughter, could help in improving the animal's homeostasis maintenance although further studies are necessary to optimize the correct dosage and period of GBs administration.

## Goji Effects on Meat Quality

Rabbit meat can be considered as a food with additional functions related to health promotion and disease prevention (104). The transformation of meat into functional food could be obtained by the addition in the rabbit's diet of functional compounds (104). Moreover, diet supplementation has also been employed to improve rabbit meat conservation by increasing its oxidative stability (105). Indeed, rabbit meat is characterized by a high unsaturated fatty acid proportion (106), which, together with the type of husbandry, make it extremely susceptible to oxidative phenomena (107, 108).

Three studies have investigated the effect of a GB-supplemented diet on rabbit meat quality. The first investigated the effect of a 30-day diet with LBP supplementation (0.1, 0.2, and 0.3%) on female and male Rex rabbits (50). No significant effects were found on any of the meat quality's evaluated parameters. In two other studies, dried GBs were added to a standard diet and administered to New Zealand White rabbits (56, 57). In the first study, male rabbits slaughtered at 91 days of age were fed with a standard diet supplemented with 0, 1, or 3% GB (56). Parameters such as color, water holding capacity (both drip and cooking losses), and tenderness were not affected by the diet, while rabbit meat from the GB groups showed improvements in the antioxidant properties such as meat pH, parameters associated with oxidative stability, and phenolic content, in a dose-dependent way. The other study was conducted on multiparous does using the same doses of GBs for 105 days (57). Meat microbiological quality analysis showed a significantly higher prevalence of *Lactobacillus* spp. in the rabbits fed with GBs. That could be a positive effect because *Lactobacillus* spp. can contrast the development of unwanted bacteria on meat. Physicochemically, the results were consistent with the previous research (56) and confirmed that GB supplementation increases the antioxidant properties of meat (57) also favoring its possibility of transformation. In addition, a sensory evaluation of rabbit meatballs coming from both groups was performed. The GB meatballs were indicated as juicier and tastier and were more appreciated by consumers than those from the control group. Moreover, after an informed session, consumers also expressed a higher interest in the purchase of GB meatballs.

Studies performed on other animal species included one that was carried out on 5-month-old piglets. Dried GBs supplemented at 1% did not affect meat quality (39). However, that result could be ascribed to the short duration of the dietary treatment (7 days). The antioxidant properties of GB have also been employed to improve the quality and conservation by being added to the final animal products. Two studies employing flavonoids extracted from *L. barbarum* leaves in different proportions (0.5, 1.0, and 1.5%) mixed with minced mutton meat indicated that they could significantly inhibit lipid oxidation and myofibrillar proteins oxidation and could therefore be utilized as a natural antioxidant for meat preservation (58, 59). The same results were found in a study conducted on horse meat products injected with 1.0% of GB extract (62). A combination of dried GBs and pumpkin powder has shown, on the cooked and smoked beef filet, the maintenance of the quality, sensory properties, color characteristics, and prevention of oxidative changes, even with a reduced amount of nitrites (60). Dried GBs were also employed as additives of a multicomponent brine on camel meat (61). *Lycium barbarum* polysaccharides and pumpkin polysaccharides had a positive effect on moisture retention, causing higher meat quality. Finally, different studies have employed *L. barbarum* extract in fish products as an antibacterial agent with encouraging results (63, 64).

Both quality and preservation of meat seem to be enhanced by the supplementation of GBs in animal diets or directly in the final product because of its antioxidant activity and enhancement action on lactic bacteria. Moreover, the consumer has a positive sensory perception of the final product and a positive image of it as a natural and healthy product, which could be used for marketing purposes.

## Conclusions and Prospects

Goji berries have proven effective in enhancing reproductive and productive performances, meat quality, immune system, and metabolic homeostasis in rabbits and other livestock species. *Lycium barbarum* fruit supplementation could represent a good strategy to produce new functional food and thus relaunch the rabbit meat sector. Goji berries could determine health benefits not only for animals but also for the consumers and have a role in optimizing production as well as in reducing the use of drugs. Despite the encouraging results, further research is needed to investigate the correct dosage and period of administration of GBs, its availability, and the economical sustainability for the preparation of supplemented feed.

## Author Contributions

DV, LM, MM, and GB: conceptualization and supervision. GC, SA, EC, OB, MCa, LM, EA, FR, and MRC: writing—original draft preparation. GC, SA, SD, AQ, MS, LM, and GB: writing—review and editing. EC, MS, MRC, and FR: visualization. All authors contributed to the article and approved the submitted version.

## Funding

This research was supported by FAR 2019 of the University of Sassari.

## Conflict of Interest

The authors declare that the research was conducted in the absence of any commercial or financial relationships that could be construed as a potential conflict of interest.

## Publisher's Note

All claims expressed in this article are solely those of the authors and do not necessarily represent those of their affiliated organizations, or those of the publisher, the editors and the reviewers. Any product that may be evaluated in this article, or claim that may be made by its manufacturer, is not guaranteed or endorsed by the publisher.
